# Methyl 2-(2,3,5-trimethyl-1,1-dioxo-2*H*-1λ^6^,2,6-thia­diazin-4-yl)benzoate

**DOI:** 10.1107/S1600536812046375

**Published:** 2012-11-17

**Authors:** Nilay Bhatt, Pralav Bhatt, Kiran Nimavat, Thavendran Govender, Hendrik G. Kruger, Glenn E. M. Maguire

**Affiliations:** a Chemistry Department, JJT University, Rajasthan, India; b School of Chemistry & Physics, University of KwaZulu-Natal, Durban 4000, South Africa; c Department of Chemistry, Government Science College, Gandhinagar, Gujarat, India; d School of Pharmacology, University of KwaZulu Natal, Westville Campus, Private Bag X54001, South Africa

## Abstract

There are two mol­ecules, *A* and *B*, in the asymmetric unit of the title compound, C_14_H_16_N_2_O_4_S, which is the first example reported in this family of compounds in which the N*sp* ^3^ atom of the thia­diazine ring is methyl­ated. The thia­diazine rings adopt shallow envelope conformations, with the S atoms displaced by 0.319 (12) and 0.182 (12) Å from the mean planes of the other ring atoms in mol­ecules *A* and *B*, respectively. The dihedral angles between the thia­diazine mean planes (excluding S) and the attached benzene rings are 86.8 (3) and 86.7 (3)° for mol­ecules *A* and *B*, respectively.

## Related literature

For synthetic background, see: Wright (1964 ▶). For a related structure, see: Bhatt *et al.* (2012 ▶). For puckering parameters, see: Cremer & Pople (1975 ▶).
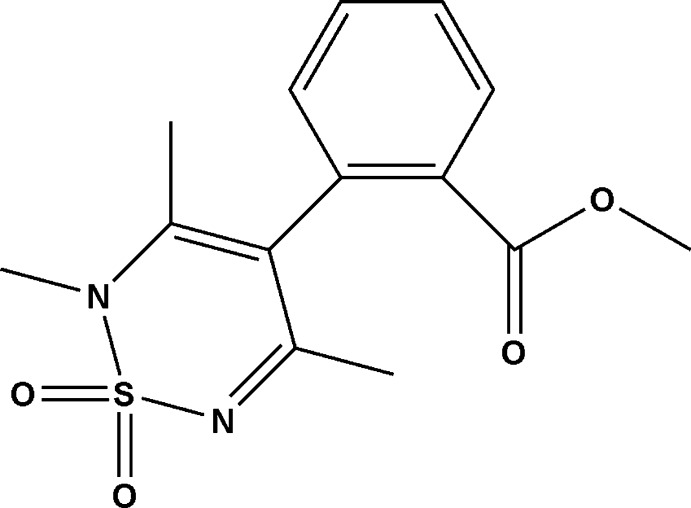


## Experimental

### 

#### Crystal data

C_14_H_16_N_2_O_4_S
*M* *_r_* = 308.35
Orthorhombic, 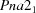

*a* = 13.5954 (3) Å
*b* = 8.0683 (2) Å
*c* = 25.9554 (7) Å
*V* = 2847.09 (12) Å^3^
*Z* = 8
Mo *K*α radiation
μ = 0.25 mm^−1^
*T* = 173 K
0.28 × 0.22 × 0.21 mm

#### Data collection

Nonius KappaCCD diffractometer
Absorption correction: multi-scan (*SADABS*; Sheldrick, 1996 ▶) *T*_min_ = 0.935, *T*_max_ = 0.950
33574 measured reflections
6094 independent reflections
4522 reflections with *I* > 2σ(*I*)
*R*_int_ = 0.057

#### Refinement

*R*[*F* ^2^ > 2σ(*F* ^2^)] = 0.037
*wR*(*F* ^2^) = 0.083
*S* = 0.99
6094 reflections
388 parameters
1 restraint
H-atom parameters constrained
Δρ_max_ = 0.21 e Å^−3^
Δρ_min_ = −0.25 e Å^−3^
Absolute structure: Flack (1983 ▶), 2751 Friedel pairs
Flack parameter: −0.06 (5)

### 

Data collection: *COLLECT* (Nonius, 2000 ▶); cell refinement: *DENZO-SMN* (Otwinowski & Minor, 1997 ▶); data reduction: *DENZO-SMN*; program(s) used to solve structure: *SHELXS97* (Sheldrick, 2008 ▶); program(s) used to refine structure: *SHELXL97* (Sheldrick, 2008 ▶); molecular graphics: *OLEX2* (Dolomanov *et al.*, 2009 ▶); software used to prepare material for publication: *SHELXL97*.

## Supplementary Material

Crystal structure: contains datablock(s) I, global. DOI: 10.1107/S1600536812046375/hb6962sup1.cif

Structure factors: contains datablock(s) I. DOI: 10.1107/S1600536812046375/hb6962Isup2.hkl

Supplementary material file. DOI: 10.1107/S1600536812046375/hb6962Isup3.cml

Additional supplementary materials:  crystallographic information; 3D view; checkCIF report

## Figures and Tables

**Fig. 1.**
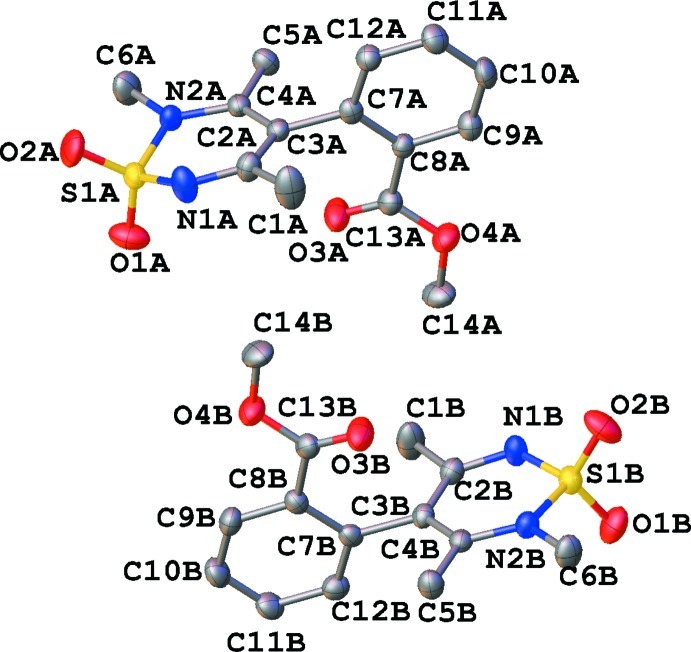
The molecular structure of the title compound with H atoms omitted for clarity. Displacement ellipsoids are drawn at 40% probability.

## supplementary crystallographic information

### Comment

The synthesis of 1,2,6-thiadiazine-1,1-dioxides derivatives was first reported using sulfamide with alpha and beta diketones (Wright, 1964). We have reported the 3,5-dimethyl based structure with an aromatic ring at position 4 of the thiadiazine ring containing an ethyl ester functional group (Bhatt *et al.*, 2012). The structure displayed intermolecular hydrogen bonding.

The title compound is also a 3,5 dimethyl based structure with an aromatic ring at the same position on the thiadiazine ring, but here the sp3 nitrogen atom is methylated. This prevents any equivalent hydrogen bonding features in the structure seen in previous examples. The is no π-π stacking or weak CH—π inteactions either in the crystal. The title compound has two isomeric forms in the crystal. The sulfur atoms S1A and S1B deviate from their ring planes (N1A/B, C2/AB, C3A/B, C4A/B, N2A/B) by 0.319 (12) Å and 0.182 (12) Å respectively (Fig. 1) and the benzene rings deviate from the planes by 86.8 (3) and 86.7 (3)°, respectively. The ester functional groups of the two isomers are not co-planar to the benzene ring planes but differ by 16.93 (4) and 16.48 (4)°, respectively.

### Experimental

2-(3, 5-dimethyl-1, 1-dioxo-2*H*-1, 2, 6-thiadiazin-4-yl) benzoic acid (4.75 g,17 mmole) was dissolved in acetone (125 ml), to this K_2_CO_3_ (11.75 g, 85 mmole) was added slowly and then stirred for 10–15 min. Methyl iodide (7.23 g, 51 mmole) was added and reaction mixture was refluxed for 3 hrs. The reaction progress was monitored by TLC using ethyl acetate/hexane (8:2,*R*_f_ = 0.8). Excess solvent was completely evaporated under vacuum. The residue was treated with conc.HCl to get 2-(2,3,5-trimethyl-1,1-dioxido-2*H*-1,2,6-thiadiazin-4-yl)benzoic acid as a white solid (4.3 g Yield: 81.9%).

2-(2,3,5-trimethyl-1,1-dioxido-2*H*-1,2,6-thiadiazin-4-yl)benzoic acid (2.94 g, 10 mmole) was dissolved in methanol (25 ml), to this solution thionyl chloride (5.95 g, 50 mmole) was added slowly and the reaction mixture was refluxed for 3 hrs. The reaction progress was monitored by TLC using ethyl acetate/hexane (8:2,*R*_f_ = 0.8). Excess solvent was completely evaporated under vacuum. The residue was purified by silica gel column using methanol/ethyl acetate (1:9) as the eluent to afford the methyl ester as a colourless solid. (1.34 g,Yield: 55%).*M*.p.= 260 K

Recrystallization using dioxane/water at room temperature afforded yellow blocks.

### Refinement

All hydrogen atoms were placed in idealized positions and refined with geometrical constraints. Flack *x* parameter is -0.0586 with e.s.d. 0.0543.

### Crystal data

**Table:**

C_14_H_16_N_2_O_4_S	*D*_x_ = 1.439 Mg m^−^^3^
*M**_r_* = 308.35	Mo *K*α radiation, λ = 0.71073 Å
Orthorhombic, *P**n**a*2_1_	Cell parameters from 33574 reflections
*a* = 13.5954 (3) Å	θ = 2.6–27.5°
*b* = 8.0683 (2) Å	µ = 0.25 mm^−^^1^
*c* = 25.9554 (7) Å	*T* = 173 K
*V* = 2847.09 (12) Å^3^	Block, yellow
*Z* = 8	0.28 × 0.22 × 0.21 mm
*F*(000) = 1296	

### Data collection

**Table:**

Nonius KappaCCD diffractometer	6094 independent reflections
Radiation source: fine-focus sealed tube	4522 reflections with *I* > 2σ(*I*)
Graphite monochromator	*R*_int_ = 0.057
1.2° φ scans and ω scans	θ_max_ = 27.5°, θ_min_ = 2.6°
Absorption correction: multi-scan (*SADABS*; Sheldrick, 1996)	*h* = −17→17
*T*_min_ = 0.935, *T*_max_ = 0.950	*k* = −10→10
33574 measured reflections	*l* = −33→30

### Refinement

**Table:**

Refinement on *F*^2^	Hydrogen site location: inferred from neighbouring sites
Least-squares matrix: full	H-atom parameters constrained
*R*[*F*^2^ > 2σ(*F*^2^)] = 0.037	*w* = 1/[σ^2^(*F*_o_^2^) + (0.0445*P*)^2^] where *P* = (*F*_o_^2^ + 2*F*_c_^2^)/3
*wR*(*F*^2^) = 0.083	(Δ/σ)_max_ < 0.001
*S* = 0.99	Δρ_max_ = 0.21 e Å^−^^3^
6094 reflections	Δρ_min_ = −0.25 e Å^−^^3^
388 parameters	Extinction correction: *SHELXL97* (Sheldrick, 2008), Fc^*^=kFc[1+0.001xFc^2^λ^3^/sin(2θ)]^-1/4^
1 restraint	Extinction coefficient: 0.0022 (4)
Primary atom site location: structure-invariant direct methods	Absolute structure: Flack (1983), 2751 Friedel pairs
Secondary atom site location: difference Fourier map	Flack parameter: −0.06 (5)

### Special details

**Table:**

Geometry. All e.s.d.'s (except the e.s.d. in the dihedral angle between two l.s. planes) are estimated using the full covariance matrix. The cell e.s.d.'s are taken into account individually in the estimation of e.s.d.'s in distances, angles and torsion angles; correlations between e.s.d.'s in cell parameters are only used when they are defined by crystal symmetry. An approximate (isotropic) treatment of cell e.s.d.'s is used for estimating e.s.d.'s involving l.s. planes.
Refinement. Refinement of *F*^2^ against ALL reflections. The weighted *R*-factor *wR* and goodness of fit *S* are based on *F*^2^, conventional *R*-factors *R* are based on *F*, with *F* set to zero for negative *F*^2^. The threshold expression of *F*^2^ > σ(*F*^2^) is used only for calculating *R*-factors(gt) *etc*. and is not relevant to the choice of reflections for refinement. *R*-factors based on *F*^2^ are statistically about twice as large as those based on *F*, and *R*- factors based on ALL data will be even larger.

### Fractional atomic coordinates and isotropic or equivalent isotropic displacement parameters (Å^2^)

**Table:**

	*x*	*y*	*z*	*U*_iso_*/*U*_eq_	
S1A	0.16819 (4)	0.24471 (7)	0.03146 (3)	0.02850 (14)	
O1A	0.17683 (12)	0.3922 (2)	0.06114 (8)	0.0473 (5)	
O2A	0.19297 (13)	0.2610 (2)	−0.02157 (7)	0.0484 (5)	
O3A	0.01809 (15)	0.1560 (2)	0.18508 (7)	0.0479 (5)	
O4A	0.01511 (13)	0.01517 (19)	0.25899 (7)	0.0390 (4)	
N1A	0.22849 (14)	0.0982 (2)	0.05644 (9)	0.0407 (6)	
N2A	0.05030 (13)	0.1839 (2)	0.03452 (8)	0.0281 (4)	
C1A	0.26014 (19)	−0.1285 (4)	0.11282 (13)	0.0533 (9)	
H1A1	0.3238	−0.1211	0.0953	0.080*	
H1A2	0.2355	−0.2424	0.1107	0.080*	
H1A3	0.2680	−0.0971	0.1491	0.080*	
C2A	0.18851 (17)	−0.0136 (3)	0.08748 (10)	0.0324 (6)	
C3A	0.08626 (17)	−0.0289 (2)	0.09642 (9)	0.0242 (5)	
C4A	0.02022 (16)	0.0661 (3)	0.06916 (9)	0.0252 (5)	
C5A	−0.08899 (15)	0.0468 (3)	0.07394 (11)	0.0315 (6)	
H5A1	−0.1181	0.1524	0.0847	0.047*	
H5A2	−0.1038	−0.0384	0.0997	0.047*	
H5A3	−0.1165	0.0138	0.0406	0.047*	
C6A	−0.01996 (19)	0.2803 (3)	0.00249 (11)	0.0439 (7)	
H6A1	−0.0556	0.2049	−0.0205	0.066*	
H6A2	0.0163	0.3619	−0.0181	0.066*	
H6A3	−0.0669	0.3377	0.0249	0.066*	
C7A	0.04993 (16)	−0.1651 (3)	0.13101 (10)	0.0254 (5)	
C8A	0.02189 (16)	−0.1410 (3)	0.18317 (9)	0.0248 (5)	
C9A	−0.00644 (17)	−0.2776 (3)	0.21258 (11)	0.0334 (6)	
H9A	−0.0260	−0.2618	0.2474	0.040*	
C10A	−0.00654 (18)	−0.4363 (3)	0.19185 (10)	0.0359 (6)	
H10A	−0.0251	−0.5284	0.2125	0.043*	
C11A	0.02038 (17)	−0.4595 (3)	0.14124 (11)	0.0343 (6)	
H11A	0.0201	−0.5677	0.1268	0.041*	
C12A	0.04773 (17)	−0.3253 (3)	0.11149 (10)	0.0299 (6)	
H12A	0.0656	−0.3431	0.0765	0.036*	
C13A	0.01886 (17)	0.0251 (3)	0.20735 (10)	0.0303 (6)	
C14A	0.0134 (2)	0.1689 (3)	0.28670 (11)	0.0429 (7)	
H14A	0.0672	0.2399	0.2747	0.064*	
H14B	0.0214	0.1471	0.3236	0.064*	
H14C	−0.0496	0.2248	0.2808	0.064*	
S1B	0.10386 (4)	0.26833 (7)	0.45180 (2)	0.02889 (15)	
O1B	0.08708 (12)	0.3200 (2)	0.50324 (7)	0.0447 (5)	
O2B	0.08897 (11)	0.09637 (19)	0.44206 (8)	0.0484 (5)	
O3B	0.24965 (14)	0.35986 (19)	0.29068 (7)	0.0485 (5)	
O4B	0.24683 (13)	0.50555 (18)	0.21771 (7)	0.0395 (5)	
N1B	0.04139 (13)	0.3758 (2)	0.41302 (9)	0.0339 (5)	
N2B	0.22197 (13)	0.3092 (2)	0.43775 (8)	0.0281 (5)	
C1B	0.00445 (19)	0.5920 (4)	0.35389 (13)	0.0504 (8)	
H1B1	0.0175	0.5786	0.3170	0.076*	
H1B2	0.0086	0.7096	0.3631	0.076*	
H1B3	−0.0616	0.5504	0.3618	0.076*	
C2B	0.07937 (18)	0.4957 (3)	0.38431 (9)	0.0285 (5)	
C3B	0.18034 (17)	0.5317 (3)	0.38014 (9)	0.0243 (5)	
C4B	0.24909 (16)	0.4372 (3)	0.40624 (9)	0.0242 (5)	
C5B	0.35705 (16)	0.4635 (3)	0.40010 (11)	0.0332 (6)	
H5B1	0.3863	0.4888	0.4337	0.050*	
H5B2	0.3686	0.5562	0.3765	0.050*	
H5B3	0.3872	0.3628	0.3861	0.050*	
C6B	0.29451 (18)	0.2085 (3)	0.46649 (11)	0.0398 (7)	
H6B1	0.3402	0.1565	0.4422	0.060*	
H6B2	0.2601	0.1224	0.4861	0.060*	
H6B3	0.3312	0.2799	0.4902	0.060*	
C7B	0.21176 (16)	0.6741 (2)	0.34682 (10)	0.0250 (5)	
C8B	0.23889 (15)	0.6576 (3)	0.29426 (10)	0.0259 (5)	
C9B	0.26085 (17)	0.7992 (3)	0.26573 (10)	0.0318 (6)	
H9B	0.2788	0.7882	0.2305	0.038*	
C10B	0.25717 (18)	0.9542 (3)	0.28739 (11)	0.0367 (6)	
H10B	0.2726	1.0490	0.2672	0.044*	
C11B	0.23110 (18)	0.9722 (3)	0.33840 (10)	0.0348 (6)	
H11B	0.2286	1.0794	0.3535	0.042*	
C12B	0.20849 (18)	0.8329 (3)	0.36763 (10)	0.0313 (6)	
H12B	0.1903	0.8465	0.4027	0.038*	
C13B	0.24545 (16)	0.4921 (3)	0.26875 (10)	0.0283 (6)	
C14B	0.2508 (2)	0.3515 (3)	0.18876 (10)	0.0433 (7)	
H14D	0.3151	0.2989	0.1939	0.065*	
H14E	0.2412	0.3748	0.1520	0.065*	
H14F	0.1989	0.2768	0.2008	0.065*	

### Atomic displacement parameters (Å^2^)

**Table:**

	*U*^11^	*U*^22^	*U*^33^	*U*^12^	*U*^13^	*U*^23^
S1A	0.0315 (3)	0.0281 (3)	0.0259 (3)	−0.0024 (3)	0.0061 (3)	0.0015 (3)
O1A	0.0388 (10)	0.0397 (10)	0.0635 (15)	−0.0022 (8)	−0.0006 (9)	−0.0202 (9)
O2A	0.0550 (11)	0.0627 (12)	0.0276 (11)	−0.0042 (9)	0.0155 (9)	0.0088 (9)
O3A	0.0854 (15)	0.0268 (10)	0.0315 (11)	−0.0043 (9)	0.0104 (10)	−0.0007 (8)
O4A	0.0536 (11)	0.0373 (10)	0.0261 (11)	0.0004 (8)	0.0042 (9)	−0.0035 (8)
N1A	0.0275 (11)	0.0427 (12)	0.0520 (16)	0.0031 (9)	0.0068 (10)	0.0163 (11)
N2A	0.0270 (10)	0.0294 (10)	0.0280 (12)	−0.0015 (8)	−0.0031 (9)	0.0067 (10)
C1A	0.0287 (14)	0.0637 (19)	0.067 (2)	0.0114 (13)	0.0020 (14)	0.0279 (16)
C2A	0.0294 (13)	0.0372 (14)	0.0305 (15)	0.0034 (11)	0.0010 (11)	0.0061 (11)
C3A	0.0271 (12)	0.0242 (11)	0.0213 (13)	−0.0004 (9)	0.0015 (10)	−0.0011 (10)
C4A	0.0295 (12)	0.0239 (12)	0.0223 (13)	−0.0019 (9)	0.0007 (10)	−0.0019 (10)
C5A	0.0271 (13)	0.0286 (12)	0.0388 (16)	−0.0015 (10)	−0.0014 (11)	0.0032 (11)
C6A	0.0427 (16)	0.0372 (14)	0.052 (2)	0.0030 (12)	−0.0096 (14)	0.0166 (13)
C7A	0.0200 (11)	0.0265 (12)	0.0297 (15)	0.0033 (9)	−0.0009 (10)	0.0040 (10)
C8A	0.0251 (11)	0.0252 (12)	0.0240 (14)	0.0013 (9)	−0.0012 (10)	0.0028 (10)
C9A	0.0333 (13)	0.0342 (13)	0.0328 (16)	−0.0006 (11)	0.0035 (11)	0.0058 (12)
C10A	0.0424 (14)	0.0282 (13)	0.0372 (17)	−0.0024 (11)	0.0017 (13)	0.0120 (12)
C11A	0.0386 (14)	0.0247 (12)	0.0396 (17)	0.0033 (10)	0.0015 (12)	0.0029 (12)
C12A	0.0328 (13)	0.0284 (13)	0.0284 (15)	0.0012 (10)	0.0023 (11)	−0.0004 (11)
C13A	0.0301 (14)	0.0330 (14)	0.0279 (16)	−0.0022 (10)	0.0037 (11)	0.0006 (11)
C14A	0.0494 (16)	0.0449 (16)	0.0342 (17)	−0.0054 (12)	0.0060 (13)	−0.0159 (12)
S1B	0.0294 (3)	0.0277 (3)	0.0295 (4)	0.0015 (2)	0.0053 (3)	0.0029 (3)
O1B	0.0433 (10)	0.0624 (12)	0.0284 (12)	0.0088 (9)	0.0098 (9)	0.0041 (10)
O2B	0.0416 (10)	0.0235 (8)	0.0800 (17)	−0.0027 (7)	0.0053 (10)	−0.0049 (9)
O3B	0.0853 (15)	0.0260 (9)	0.0342 (12)	0.0006 (10)	0.0057 (11)	0.0002 (8)
O4B	0.0579 (12)	0.0349 (10)	0.0257 (12)	−0.0001 (8)	0.0039 (8)	−0.0017 (8)
N1B	0.0273 (11)	0.0402 (12)	0.0342 (13)	−0.0019 (9)	0.0021 (9)	0.0113 (10)
N2B	0.0282 (10)	0.0297 (9)	0.0266 (12)	0.0022 (8)	−0.0006 (9)	0.0040 (9)
C1B	0.0318 (14)	0.0645 (18)	0.055 (2)	−0.0014 (13)	−0.0074 (14)	0.0273 (15)
C2B	0.0301 (12)	0.0297 (12)	0.0257 (14)	0.0033 (10)	−0.0003 (10)	0.0019 (10)
C3B	0.0283 (12)	0.0239 (11)	0.0208 (13)	0.0020 (9)	0.0031 (10)	−0.0003 (10)
C4B	0.0281 (11)	0.0217 (11)	0.0230 (13)	−0.0006 (9)	0.0002 (10)	−0.0010 (10)
C5B	0.0277 (12)	0.0356 (13)	0.0364 (16)	0.0004 (10)	0.0000 (11)	0.0068 (11)
C6B	0.0340 (13)	0.0436 (13)	0.0417 (19)	0.0071 (11)	−0.0039 (12)	0.0145 (13)
C7B	0.0215 (11)	0.0239 (12)	0.0295 (15)	−0.0005 (9)	−0.0045 (10)	0.0020 (10)
C8B	0.0238 (12)	0.0240 (12)	0.0299 (15)	−0.0008 (9)	−0.0002 (10)	0.0024 (10)
C9B	0.0350 (13)	0.0350 (13)	0.0254 (15)	0.0004 (10)	0.0011 (11)	0.0061 (11)
C10B	0.0433 (15)	0.0256 (13)	0.0413 (18)	−0.0019 (11)	−0.0004 (13)	0.0077 (12)
C11B	0.0405 (14)	0.0223 (12)	0.0415 (18)	0.0003 (10)	−0.0050 (12)	0.0019 (11)
C12B	0.0332 (13)	0.0296 (13)	0.0311 (16)	0.0022 (11)	−0.0039 (11)	−0.0003 (11)
C13B	0.0261 (12)	0.0324 (14)	0.0265 (15)	−0.0028 (10)	0.0026 (11)	0.0000 (11)
C14B	0.0547 (17)	0.0465 (16)	0.0286 (17)	−0.0063 (12)	0.0051 (13)	−0.0135 (12)

### Geometric parameters (Å, º)

**Table:**

S1A—O1A	1.4223 (17)	S1B—O1B	1.4171 (18)
S1A—O2A	1.4233 (19)	S1B—O2B	1.4247 (17)
S1A—N1A	1.578 (2)	S1B—N1B	1.577 (2)
S1A—N2A	1.6781 (18)	S1B—N2B	1.6793 (19)
O3A—C13A	1.204 (3)	O3B—C13B	1.211 (3)
O4A—C13A	1.344 (3)	O4B—C13B	1.329 (3)
O4A—C14A	1.434 (3)	O4B—C14B	1.453 (3)
N1A—C2A	1.326 (3)	N1B—C2B	1.326 (3)
N2A—C4A	1.371 (3)	N2B—C4B	1.368 (3)
N2A—C6A	1.486 (3)	N2B—C6B	1.480 (3)
C1A—C2A	1.496 (3)	C1B—C2B	1.505 (3)
C1A—H1A1	0.9800	C1B—H1B1	0.9800
C1A—H1A2	0.9800	C1B—H1B2	0.9800
C1A—H1A3	0.9800	C1B—H1B3	0.9800
C2A—C3A	1.415 (3)	C2B—C3B	1.407 (3)
C3A—C4A	1.377 (3)	C3B—C4B	1.383 (3)
C3A—C7A	1.502 (3)	C3B—C7B	1.500 (3)
C4A—C5A	1.498 (3)	C4B—C5B	1.492 (3)
C5A—H5A1	0.9800	C5B—H5B1	0.9800
C5A—H5A2	0.9800	C5B—H5B2	0.9800
C5A—H5A3	0.9800	C5B—H5B3	0.9800
C6A—H6A1	0.9800	C6B—H6B1	0.9800
C6A—H6A2	0.9800	C6B—H6B2	0.9800
C6A—H6A3	0.9800	C6B—H6B3	0.9800
C7A—C12A	1.388 (3)	C7B—C12B	1.391 (3)
C7A—C8A	1.420 (3)	C7B—C8B	1.419 (3)
C8A—C9A	1.395 (3)	C8B—C9B	1.394 (3)
C8A—C13A	1.481 (3)	C8B—C13B	1.493 (3)
C9A—C10A	1.389 (4)	C9B—C10B	1.372 (3)
C9A—H9A	0.9500	C9B—H9B	0.9500
C10A—C11A	1.376 (3)	C10B—C11B	1.378 (4)
C10A—H10A	0.9500	C10B—H10B	0.9500
C11A—C12A	1.381 (3)	C11B—C12B	1.391 (3)
C11A—H11A	0.9500	C11B—H11B	0.9500
C12A—H12A	0.9500	C12B—H12B	0.9500
C14A—H14A	0.9800	C14B—H14D	0.9800
C14A—H14B	0.9800	C14B—H14E	0.9800
C14A—H14C	0.9800	C14B—H14F	0.9800
			
O1A—S1A—O2A	115.27 (12)	O1B—S1B—O2B	115.51 (12)
O1A—S1A—N1A	111.17 (13)	O1B—S1B—N1B	110.67 (11)
O2A—S1A—N1A	110.10 (11)	O2B—S1B—N1B	110.22 (11)
O1A—S1A—N2A	107.33 (10)	O1B—S1B—N2B	107.50 (10)
O2A—S1A—N2A	107.39 (11)	O2B—S1B—N2B	106.78 (9)
N1A—S1A—N2A	104.94 (10)	N1B—S1B—N2B	105.57 (10)
C13A—O4A—C14A	116.69 (19)	C13B—O4B—C14B	116.48 (18)
C2A—N1A—S1A	123.12 (16)	C2B—N1B—S1B	123.38 (17)
C4A—N2A—C6A	122.55 (19)	C4B—N2B—C6B	122.43 (19)
C4A—N2A—S1A	121.25 (15)	C4B—N2B—S1B	122.41 (15)
C6A—N2A—S1A	115.74 (15)	C6B—N2B—S1B	114.83 (16)
C2A—C1A—H1A1	109.5	C2B—C1B—H1B1	109.5
C2A—C1A—H1A2	109.5	C2B—C1B—H1B2	109.5
H1A1—C1A—H1A2	109.5	H1B1—C1B—H1B2	109.5
C2A—C1A—H1A3	109.5	C2B—C1B—H1B3	109.5
H1A1—C1A—H1A3	109.5	H1B1—C1B—H1B3	109.5
H1A2—C1A—H1A3	109.5	H1B2—C1B—H1B3	109.5
N1A—C2A—C3A	124.2 (2)	N1B—C2B—C3B	125.0 (2)
N1A—C2A—C1A	114.9 (2)	N1B—C2B—C1B	114.1 (2)
C3A—C2A—C1A	120.9 (2)	C3B—C2B—C1B	120.9 (2)
C4A—C3A—C2A	120.5 (2)	C4B—C3B—C2B	120.5 (2)
C4A—C3A—C7A	120.01 (19)	C4B—C3B—C7B	120.79 (19)
C2A—C3A—C7A	119.01 (19)	C2B—C3B—C7B	118.7 (2)
N2A—C4A—C3A	121.93 (19)	N2B—C4B—C3B	121.77 (19)
N2A—C4A—C5A	114.97 (19)	N2B—C4B—C5B	115.88 (19)
C3A—C4A—C5A	123.1 (2)	C3B—C4B—C5B	122.3 (2)
C4A—C5A—H5A1	109.5	C4B—C5B—H5B1	109.5
C4A—C5A—H5A2	109.5	C4B—C5B—H5B2	109.5
H5A1—C5A—H5A2	109.5	H5B1—C5B—H5B2	109.5
C4A—C5A—H5A3	109.5	C4B—C5B—H5B3	109.5
H5A1—C5A—H5A3	109.5	H5B1—C5B—H5B3	109.5
H5A2—C5A—H5A3	109.5	H5B2—C5B—H5B3	109.5
N2A—C6A—H6A1	109.5	N2B—C6B—H6B1	109.5
N2A—C6A—H6A2	109.5	N2B—C6B—H6B2	109.5
H6A1—C6A—H6A2	109.5	H6B1—C6B—H6B2	109.5
N2A—C6A—H6A3	109.5	N2B—C6B—H6B3	109.5
H6A1—C6A—H6A3	109.5	H6B1—C6B—H6B3	109.5
H6A2—C6A—H6A3	109.5	H6B2—C6B—H6B3	109.5
C12A—C7A—C8A	118.0 (2)	C12B—C7B—C8B	117.9 (2)
C12A—C7A—C3A	118.0 (2)	C12B—C7B—C3B	118.2 (2)
C8A—C7A—C3A	123.9 (2)	C8B—C7B—C3B	123.80 (19)
C9A—C8A—C7A	119.2 (2)	C9B—C8B—C7B	119.3 (2)
C9A—C8A—C13A	118.4 (2)	C9B—C8B—C13B	119.0 (2)
C7A—C8A—C13A	122.4 (2)	C7B—C8B—C13B	121.7 (2)
C10A—C9A—C8A	121.1 (2)	C10B—C9B—C8B	121.4 (2)
C10A—C9A—H9A	119.4	C10B—C9B—H9B	119.3
C8A—C9A—H9A	119.4	C8B—C9B—H9B	119.3
C11A—C10A—C9A	119.6 (2)	C9B—C10B—C11B	119.9 (2)
C11A—C10A—H10A	120.2	C9B—C10B—H10B	120.0
C9A—C10A—H10A	120.2	C11B—C10B—H10B	120.0
C10A—C11A—C12A	119.9 (2)	C10B—C11B—C12B	119.7 (2)
C10A—C11A—H11A	120.0	C10B—C11B—H11B	120.2
C12A—C11A—H11A	120.0	C12B—C11B—H11B	120.2
C11A—C12A—C7A	122.1 (2)	C7B—C12B—C11B	121.7 (2)
C11A—C12A—H12A	118.9	C7B—C12B—H12B	119.1
C7A—C12A—H12A	118.9	C11B—C12B—H12B	119.1
O3A—C13A—O4A	122.1 (2)	O3B—C13B—O4B	122.7 (2)
O3A—C13A—C8A	126.2 (2)	O3B—C13B—C8B	125.6 (2)
O4A—C13A—C8A	111.7 (2)	O4B—C13B—C8B	111.7 (2)
O4A—C14A—H14A	109.5	O4B—C14B—H14D	109.5
O4A—C14A—H14B	109.5	O4B—C14B—H14E	109.5
H14A—C14A—H14B	109.5	H14D—C14B—H14E	109.5
O4A—C14A—H14C	109.5	O4B—C14B—H14F	109.5
H14A—C14A—H14C	109.5	H14D—C14B—H14F	109.5
H14B—C14A—H14C	109.5	H14E—C14B—H14F	109.5
			
O1A—S1A—N1A—C2A	95.6 (2)	O1B—S1B—N1B—C2B	103.6 (2)
O2A—S1A—N1A—C2A	−135.4 (2)	O2B—S1B—N1B—C2B	−127.4 (2)
N2A—S1A—N1A—C2A	−20.1 (3)	N2B—S1B—N1B—C2B	−12.5 (2)
O1A—S1A—N2A—C4A	−96.67 (19)	O1B—S1B—N2B—C4B	−106.12 (19)
O2A—S1A—N2A—C4A	138.84 (18)	O2B—S1B—N2B—C4B	129.36 (19)
N1A—S1A—N2A—C4A	21.7 (2)	N1B—S1B—N2B—C4B	12.1 (2)
O1A—S1A—N2A—C6A	75.8 (2)	O1B—S1B—N2B—C6B	67.42 (19)
O2A—S1A—N2A—C6A	−48.72 (19)	O2B—S1B—N2B—C6B	−57.1 (2)
N1A—S1A—N2A—C6A	−165.88 (19)	N1B—S1B—N2B—C6B	−174.41 (18)
S1A—N1A—C2A—C3A	8.8 (4)	S1B—N1B—C2B—C3B	7.2 (4)
S1A—N1A—C2A—C1A	−172.1 (2)	S1B—N1B—C2B—C1B	−174.6 (2)
N1A—C2A—C3A—C4A	5.5 (4)	N1B—C2B—C3B—C4B	1.3 (4)
C1A—C2A—C3A—C4A	−173.6 (2)	C1B—C2B—C3B—C4B	−176.7 (2)
N1A—C2A—C3A—C7A	177.7 (2)	N1B—C2B—C3B—C7B	−178.7 (2)
C1A—C2A—C3A—C7A	−1.3 (3)	C1B—C2B—C3B—C7B	3.2 (3)
C6A—N2A—C4A—C3A	176.2 (2)	C6B—N2B—C4B—C3B	−179.1 (2)
S1A—N2A—C4A—C3A	−11.9 (3)	S1B—N2B—C4B—C3B	−6.1 (3)
C6A—N2A—C4A—C5A	−2.4 (3)	C6B—N2B—C4B—C5B	3.4 (3)
S1A—N2A—C4A—C5A	169.47 (16)	S1B—N2B—C4B—C5B	176.44 (17)
C2A—C3A—C4A—N2A	−3.3 (3)	C2B—C3B—C4B—N2B	−1.6 (3)
C7A—C3A—C4A—N2A	−175.4 (2)	C7B—C3B—C4B—N2B	178.5 (2)
C2A—C3A—C4A—C5A	175.3 (2)	C2B—C3B—C4B—C5B	175.7 (2)
C7A—C3A—C4A—C5A	3.1 (3)	C7B—C3B—C4B—C5B	−4.2 (3)
C4A—C3A—C7A—C12A	96.2 (3)	C4B—C3B—C7B—C12B	−97.4 (3)
C2A—C3A—C7A—C12A	−76.1 (3)	C2B—C3B—C7B—C12B	82.7 (3)
C4A—C3A—C7A—C8A	−86.8 (3)	C4B—C3B—C7B—C8B	86.7 (3)
C2A—C3A—C7A—C8A	100.9 (3)	C2B—C3B—C7B—C8B	−93.2 (3)
C12A—C7A—C8A—C9A	0.1 (3)	C12B—C7B—C8B—C9B	−0.1 (3)
C3A—C7A—C8A—C9A	−177.0 (2)	C3B—C7B—C8B—C9B	175.9 (2)
C12A—C7A—C8A—C13A	−178.2 (2)	C12B—C7B—C8B—C13B	179.3 (2)
C3A—C7A—C8A—C13A	4.7 (3)	C3B—C7B—C8B—C13B	−4.7 (3)
C7A—C8A—C9A—C10A	0.7 (3)	C7B—C8B—C9B—C10B	0.2 (3)
C13A—C8A—C9A—C10A	179.1 (2)	C13B—C8B—C9B—C10B	−179.2 (2)
C8A—C9A—C10A—C11A	−0.9 (4)	C8B—C9B—C10B—C11B	−0.1 (4)
C9A—C10A—C11A—C12A	0.3 (3)	C9B—C10B—C11B—C12B	−0.1 (4)
C10A—C11A—C12A—C7A	0.5 (3)	C8B—C7B—C12B—C11B	−0.1 (3)
C8A—C7A—C12A—C11A	−0.7 (3)	C3B—C7B—C12B—C11B	−176.3 (2)
C3A—C7A—C12A—C11A	176.6 (2)	C10B—C11B—C12B—C7B	0.2 (4)
C14A—O4A—C13A—O3A	−2.0 (3)	C14B—O4B—C13B—O3B	1.7 (3)
C14A—O4A—C13A—C8A	179.1 (2)	C14B—O4B—C13B—C8B	−178.6 (2)
C9A—C8A—C13A—O3A	−161.3 (2)	C9B—C8B—C13B—O3B	163.0 (2)
C7A—C8A—C13A—O3A	16.9 (4)	C7B—C8B—C13B—O3B	−16.5 (4)
C9A—C8A—C13A—O4A	17.5 (3)	C9B—C8B—C13B—O4B	−16.7 (3)
C7A—C8A—C13A—O4A	−164.2 (2)	C7B—C8B—C13B—O4B	163.9 (2)

## References

[bb1] Bhatt, N., Bhatt, P., Vyas, K. B., Nimavat, K., Govender, T., Kruger, H. G. & Maguire, G. E. M. (2012). *Acta Cryst.* E**68**, o2160. 10.1107/S1600536812024907 PMC3393967 22798832

[bb2] Cremer, D. & Pople, J. A. (1975). *J. Am. Chem. Soc.* **97**, 1354–1358.

[bb3] Dolomanov, O. V., Bourhis, L. J., Gildea, R. J., Howard, J. A. K. & Puschmann, H. (2009). *J. Appl. Cryst.* **42**, 339–341.

[bb4] Flack, H. D. (1983). *Acta Cryst.* A**39**, 876–881.

[bb5] Nonius (2000). *COLLECT* Nonius BV, Delft, The Netherlands.

[bb6] Otwinowski, Z. & Minor, W. (1997). *Methods in Enzymology*, Vol. 276, *Macromolecular Crystallography*, Part A, edited by C. W. Carter Jr & R. M. Sweet, pp. 307–326. New York: Academic Press.

[bb7] Sheldrick, G. M. (1996). *SADABS* University of Göttingen, Germany.

[bb8] Sheldrick, G. M. (2008). *Acta Cryst.* A**64**, 112–122. 10.1107/S0108767307043930 18156677

[bb9] Wright, J. B. (1964). *J. Org. Chem.* **29**, 1905–1909.

